# Supplementary material for: Endogenous hydrogen peroxide positively regulates secretion of a gut-derived peptide in neuroendocrine potentiation of the oxidative stress response in Caenorhabditis elegans
Source: eLife. 2024 Dec 5;13:RP97503. doi: 10.7554/eLife.97503 (PMC11620748; doi:10.7554/eLife.97503)
Supplement: Supplementary file 1. [file elife-97503-supp1.docx]

| **Strain** | **Genotype** | **Source** |
| --- | --- | --- |
| N2 | wild type Bristol strain | *Caenorhabditis* Genetics Center (CGC) |
| OJ6555 | *flp-1(ok2811) IV* | CGC |
| OJ5490 | *flp-2(ok3351) X* | CGC |
| OJ10228 | *flp-1(ok2811);flp-2(ok3351)* | this paper |
| OJ7466 | *aex-4(sa22) X* | CGC |
| VC127 | *pkc-2(ok328) X* | CGC |
| OJ3614 | *vjIs150[pJQ60(Pttx-3::flp-1::Venus)]* | (Jia and Sieburth 2021), this paper |
| OJ5616 | *aex-5(sa23);vjIs150* | this paper |
| OJ5780 | *vjEx1748[pJQ298(Prab-3::aex-5 cDNA)];aex-5(sa23);vjIs150* | this paper |
| OJ5785 | *vjEx1753[pJQ299(Pges-1::aex-5 cDNA)];aex-5(sa23);vjIs150* | this paper |
| OJ6334 | *vjEx1753;aex-5(sa23);flp-2(ok3351);vjIs150* | this paper |
| OJ5264 | *flp-2(ok3351);vjIs150* | this paper |
| OJ8818 | *vjEx2882[pJQ366(Prab-3::flp-2 gDNA)];flp-2(ok3351);vjIs150* | this paper |
| OJ8813 | *vjEx2877[pJQ302(Pges-1::flp-2 gDNA)];flp-2(ok3511);vjIs150* | this paper |
| OJ10229 | *vjEx2877;vjIs150* | this paper |
| CL2166 | *dvIs19[pAF15(Pgst-4::GFP::NLS)]* |  |
| OJ2547 | *flp-1(ok2811);dvIs19* | (Jia and Sieburth 2021), this paper |
| OJ10230 | *flp-2(ok3351);dvIs19* | this paper |
| OJ6544 | *flp-1(ok2811);flp-2(ok3511);dvIs19* | this paper |
| OJ10231 | *vjEx2877;dvIs19* | this paper |
| OJ10232 | *vjEx2877;flp-1(ok2281);dvIs19* | this paper |
| OJ5888 | *aex-1(sa9);vjIs150* | this paper |
| OJ5890 | *aex-3(js815);vjIs150* | this paper |
| OJ5891 | *aex-4(sa22);vIs150* | this paper |
| OJ5892 | *aex-6(sa24);vjIs150* | this paper |
| OJ5615 | *nlp-40(tm4085);vjIs150* | this paper |
| OJ5889 | *aex-2(sa3);vjIs150* | this paper |
| OJ6405 | *vjEx2035[pJQ305(Pges-1::flp-2 gDNA::Venus)]* | this paper |
| OJ9469 | *vjEx3069[pDY10(Pges-1::aex-5::mTur2)];vjEx2035* | this paper |
| OJ6409 | *aex-4(sa22);vjEx2035* | this paper |
| OJ8345 | *aex-6(sa24);vjEx2035* | this paper |
| OJ6641 | *flp-1(ok2811);vjEx2035* | this paper |
| OJ1002 | *vjIs40[pDS292(Pnlp-40::nlp-40::Venus)]* | this paper |
| OJ10237 | *vjEx3263[pJQ370(Pges-1::nlp-27 gDNA::Venus)]* | this paper |
| OJ9567 | *vjEx3062[pDY14(Pges-1::nlp-40::mTur2)];vjEx2035* | this paper |
| OJ9797 | *sod-1(tm783);vjEx2035* | this paper |
| OJ8588 | *vjEx2814[pJQ419(Pges-1::sod-1b cDNA)];sod-1(tm783);vjEx2035* | this paper |
| OJ8341 | *sod-3(tm760);vjEx0235* | this paper |
| OJ8933 | *vjEx2910[pJQ389(Pges-1::sod-3 cDNA)];sod-3(tm760);vjEx2035* | this paper |
| OJ9106 | *vjEx2973[pJQ408(Pges-1::sod-3(∆MLS) cDNA)];sod-3(tm760);vjEx2035* | this paper |
| OJ10234 | *sod-1(tm783);sod-3(tm760);vjEx2035* | this paper |
| OJ10243 | *vjEx3266[pJQ420(Pges-1::sod-1b cDNA::GFP)]* | this paper |
| OJ9141 | *vjEx2993[pJQ407(Pges-1::sod-3 cDNA::GFP)]* | this paper |
| OJ9144 | *vjEx2996[pJQ409(Pges-1::sod-3(∆MLS) cDNA::GFP)]* | this paper |
| OJ9230 | *vjEx3020[pJQ383(Pges-1::MLS::HyPer7)]* | this paper |
| OJ9196 | *vjEx3014[pJQ411(Pges-1::tomm-20::HyPer7)]* | this paper |
| OJ9281 | *sod-1(tm783);vjEx3020* | this paper |
| OJ9259 | *sod-3(tm760);vjEx3020* | this paper |
| OJ10244 | *sod-1(tm783);sod-3(tm760);vjEx3020* | this paper |
| OJ9795 | *sod-1(tm783);vjEx3014* | this paper |
| OJ9280 | *sod-3(tm760);vjEx3014* | this paper |
| OJ10245 | *sod-1(tm783);sod-3(tm760);vjEx3014* | this paper |
| OJ10238 | *sod-2(ok1030);vjEx2035* | this paper |
| OJ10239 | *sod-4(gk101);vjEx2035* | this paper |
| OJ10240 | *sod-5(tm1146);vjEx2035* | this paper |
| OJ8991 | *prdx-2(gk169);vjEx2035* | this paper |
| OJ10251 | *prdx-2b(vj380);vjEx2035* | this paper |
| OJ8996 | *vjEx2926[pJQ381(Pges-1::prdx-2b cDNA)];prdx-2(gk169);vjEx2035* | this paper |
| OJ9249 | *trx-3(tm2820);vjEx2035* | this paper |
| OJ9496 | *vjEx3091[pJQ422(Pges-1::trx-3 cDNA)];trx-3(tm2820);vjEx2035* | this paper |
| OJ10252 | *trx-3(tm2820);sod-1(tm783);vjEx2035* | this paper |
| OJ10253 | *trx-3(tm2820);sod-3(tm760);vjEx2035* | this paper |
| OJ9237 | *prdx-2(gk169);vjEx3020* | this paper |
| OJ10247 | *prdx-2b(vj380);vjEx3020* | this paper |
| OJ10249 | *trx-3(tm2820);vjEx3020* | this paper |
| OJ10246 | *prdx-2(gk169);vjEx3014* | this paper |
| OJ10248 | *prdx-2b(vj380);vjEx3014* | this paper |
| OJ10250 | *trx-3(tm2820);vjEx3014* | this paper |
| OJ10254 | *prdx-2b(vj380);dvIs19* | this paper |
| OJ10255 | *prdx-2b(vj380);flp-2(tm3351);dvIs19* | this paper |
| OJ10256 | *prdx-3(gk529);vjEx2035* | this paper |
| OJ10258 | *vjEx3268[pJQ380(Pges-1::prdx-2a cDNA)];prdx-2(gk169);vjEx2035* | this paper |
| OJ10260 | *vjEx3270[pJQ399(Pges-1::prdx-2c cDNA)];prdx-2(gk169);vjEx2035* | this paper |
| OJ9250 | *prdx-2b(vj380);sod-3(tm760);vjEx2035* | this paper |
| OJ9682 | *pkc-2(ok328);vjEx2035* | this paper |
| OJ8682 | *vjEx2828[pJQ376(Pges-1::pkc-2b cDNA)];pkc-2(ok328);vjEx2035* | this paper |
| OJ9657 | *vjEx3131[pJQ446(Pges-1::pkc-2(K375R) cDNA)];pkc-2(ok328);vjEx2035* | this paper |
| OJ10279 | *pkc-2(ok328);vjEx3020* | this paper |
| OJ10280 | *pkc-2(ok328);vjEx3014* | this paper |
| OJ8939 | *prdx-2(gk169);pkc-2(ok328);vjEx2035* | this paper |
| OJ10278 | *pkc-1(nj3);vjEx2035* | this paper |
| OJ9863 | *egl-8(sa47);vjEx2035* | this paper |
| OJ10281 | *egl-8(sa47);vjEx3020* | this paper |
| OJ10282 | *egl-8(sa47);vjEx3014* | this paper |
| OJ10263 | *dgk-2(gk124);vjEx2035* | this paper |
| OJ10264 | *vjEx327[pJQ460(Pges-1::dgk-2a cDNA)];dgk-2(gk124);vjEx2035* | this paper |
| OJ10266 | *dgk-2(gk124);pkc-2(ok328);vjEx2035* | this paper |
| OJ10283 | *dgk-2(gk124);vjEx3020* | this paper |
| OJ10284 | *dgk-2(gk124);vjEx3014* | this paper |
| OJ9809 | *plc-2(ok1761);vjEx2035* | this paper |
| OJ9028 | *vjEx2936[pJQ382(Pttx-3::MLS::HyPer7)]* | this paper |
| OJ10595 | *vjEx2936;flp-2(ok3351)* | this paper |
